# London Orphan Asylum, Watford

**Published:** 1905-01-21

**Authors:** 


### LONDON ORPHAN ASYLUM, WATFORD.

THE Secretary of the London Orphan Asylum, Watford, writes: Sir, the attention of my board of managers has been directed to the article appearing in your issue of December 31st relating to the arrangements for the treatment of the sick at the Infant Orphan Asylum, and to the relations between the medical officers of that institution and the board of management, as to which it would be out of place for me to offer any opinion; but I may, perhaps, be allowed to refer to the latter part of your article, in which you state that

> The controversy at that institution has had one good effect. The London Orphan Asylum, recognising the danger of inadequately providing against epidemic, has taken advantage of the recommendations ignored by the Infant Orphan Asylum, and set its house in order without delay.

This is not quite in accord with the facts. My board's relations with their medical staff and consultants have all along been most cordial and unanimous, and the arrangements at the institution as bearing on the health of the children have always been such as to meet with the Medical Officer's approval, and everything relating to the welfare of the inmates has been carried out strictly in accordance with the views of the doctors. The only change that we have made since the question affecting the Infant Orphan Asylum was raised is that the Infirmary has been placed under the charge of a permanent certificated nurse and a probationer, instead of a matron with the help of trained nurses when needed. This had for some time previously been contemplated.

.˙. We cannot understand how the London Orphan Asylum authorities can interpret our article as suggesting that any friction with the medical staff existed at their institution. We neither mentioned or suggested such a thing. The sole object of our article was to give credit where credit is due, and we very much regret that our motive has been misconstrued.—ED. THE HOSPITAL.

